# Synthesis and characterization of a new ZIF-67@MgAl_2_O_4_ nanocomposite and its adsorption behaviour†

**DOI:** 10.1039/d1ra01056e

**Published:** 2021-04-08

**Authors:** Mehdi Davoodi, Fatemeh Davar, Mohammad R. Rezayat, Mohammad T. Jafari, Mehdi Bazarganipour, Ahmed Esmail Shalan

**Affiliations:** Department of Chemistry, Isfahan University of Technology Isfahan 84156-83111 Iran davar@cc.iut.ac.ir; BCMaterials, Basque Center for Materials, Applications and Nanostructures Martina Casiano, UPV/EHU Science Park, Barrio Sarriena s/n Leioa 48940 Spain a.shalan133@gmail.com ahmed.shalan@bcmaterials.net; Central Metallurgical Research and Development Institute (CMRDI) P.O. Box 87, Helwan Cairo 11421 Egypt

## Abstract

Fabricating suitable adsorbents with low-cost and high efficiency extraction for measurement of very small amounts of agricultural pesticides in food and water is playing a vital key role in personal and environmental health. Here, a new composite of zeolitic imidazolate framework-67@magnesium aluminate spinel (ZIF-67@MgAl_2_O_4_) has been fabricated by a simple method at room temperature with different weight ratios. Several techniques such as FE-SEM, BET, XRD, and TGA have been used to confirm the structural characterization of the obtained materials. The obtained ZIF-67@MgAl_2_O_4_ was utilized as an adsorbent in the solid phase microextraction technique to extract and preconcentrate the herbicide molinate (as an analyte) in aqueous solution. Corona discharge ionization-ion mobility spectrometry (CD-IMS) was applied for quantification of the analyte molecules. Extraction temperature, extraction time, stirring rate, and sample pH as the main parameters that affected the extraction proficiency were chosen and considered. Under optimal conditions, the linear dynamic range (LDR) of the various concentrations of the molinate and correlation coefficient were 10.0–100.0 μg L^−1^ and 0.9961, respectively. The limit of quantification (LOQ) and method detection limit (MDL) were 10.0 μg L^−1^ and 3.0 μg L^−1^, respectively. The relative standard deviation (RSD) of the ZIF-67@MgAl_2_O_4_ for extracting the molinate molecules (molinate concentration; 50 μg L^−1^) was calculated to be 4% and the enrichment factor (EF) was ∼5.

## Abstract 2

New nanocomposite of zeolitic imidazolate framework-67@magnesium aluminate spinel (ZIF-67@MgAl_2_O_4_) has been fabricated by a simple method at room temperature with different weight ratios.

## Graphical abstract

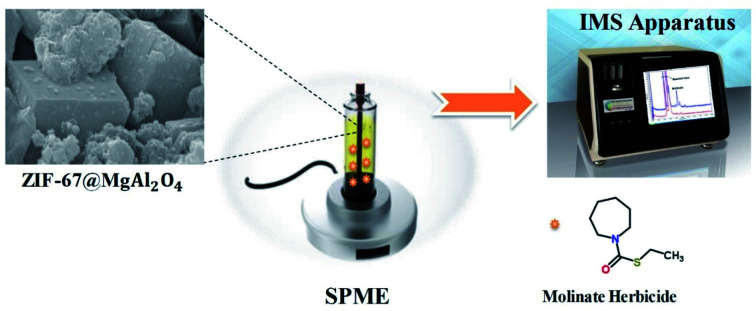

## 1. Introduction

Pesticide contamination is one of the significant global issues that humans grapple with. Therefore, extraction and measurement of agricultural pesticides by instrumental methods in food and water plays a key role in personal health and prevention of environmental problems.^1,2^ In 1970, ion mobility spectrometry (IMS) was first invented by Karasek and Cohen.^3^ The IMS technique was rapidly developed due to its advantages including high sensitivity, rapidity, and simplicity as a spectroscopy technique. The production of ions in the gas phase and the mobility of these ions in a weak electric field is the base mechanism of the IMS system. This technique is widely used for identification of various compounds, such as explosive materials, chemical gases, poisonous materials (herbicide, pesticide, and insecticide), and abuse and clinical drugs.^4^

Magnesium aluminate (MgAl_2_O_4_) is one of the furthermost significant oxide spinel and ceramic materials that has desirable mechanical properties and wide range of applications even if affected with very high temperatures.^5,6^ These applications originate from high melting point, high acid and base resistance, unique optical properties, low dielectric constant and high mechanical strength at both room temperature and high temperature.^7–9^ In a number of applications, particularly adsorption, the synthesis of the MgAl_2_O_4_ spinel has attracted a lot of attention because of its features like large surface area, little crystallite size, additional active sites, least particle agglomeration, wettability and suitable energy gap.^10–12^

Metal–organic frameworks (MOFs) recognized as a category of porous crystalline materials, have been used in many scientific researches,^13–15^ like electronic devices, adsorption, gas storage, sensors, catalysts, biomedical, separation, and luminescence applications.^16–18^ These various applications are originated from inherent features of MOFs, like permanent porosity, uniform, tunable pore size, large crystallization, tunable organic ligands, high pore volume, solvent resistance, and wide surface area.^19,20^ Zeolitic imidazolate frameworks (ZIFs) are known as a particular and novel class of metal–organic frameworks potentially including bridging organic linkers and metal ions with inherent porosity also excellent thermal and chemical stability.^21,22^ Altered uses of ZIF nanostructures are extremely affected by considering size and various morphology of the prepared materials.^23^ Different parameters like molar ratio of reactants, solvent and temperature have been able to potentially influence the size and morphology of the prepared ZIF nanostructures.^24,25^ Recently, the fabricating of new composites based on MOF in order to improve the properties of these compounds has gained an increasing amount of attention in scientific researches such as, MOF/MOF microporous,^26^ MOF/Fe_3_O_4_ microporous,^27^ ZIF-8/ZIF-67 core–shell^28^ and SiO_2_/MOF nanocomposites.^29^

In this study, a new composite of ZIF-67@MgAl_2_O_4_ was successfully prepared *via* an unpretentious procedure at room temperature with different weight proportions. The ZIF-67@MgAl_2_O_4_ nanocomposite provides many advantages such as excellent mechanical, thermal and chemical stability, additional active sites, wettability, high acid and base resistance, unique optical properties, polarity, tunable organic ligands and hydrophobicity. This composite can potentially be applied in a wide range of applications even up to very high temperatures. For characterization of ZIF-67@MgAl_2_O_4_ nanocomposite, several techniques including FE-SEM, BET, XRD, ICP, FT-IR, and TGA were used. Then, the influence of weight ratio was studied on the morphology of the ZIF-67@MgAl_2_O_4_ nanocomposites. To study the capability of the ZIF-67@MgAl_2_O_4_ as an adsorbent, solid phase microextraction (SPME) technique was utilized for preconcentration of the herbicide molinate (as analyte) in the aqueous solution. Furthermore, corona discharge ionization-ion mobility spectrometry (CD-IMS) was utilized to detect the analyte molecules. For increasing the preconcentration of the analyte from aqueous solution, some essential factors on the extraction efficiency were selected, studied and optimized. The attained composite displays a respectable way to adsorb undesirable constituents in water for environmental health applications.

## 2. Experimental part

### 2.1. Materials

All reagents are applied as received from the companies without more purification. Aluminum nitrate (Al(NO_3_)_3_·9H_2_O), magnesium nitrate (Mg(NO_3_)_2_·6H_2_O), succinic acid (C_4_H_6_O_4_) (Merck Company) and diethylene glycol (Dae-Jung Company) were used to prepare MgAl_2_O_4_ spinel. Cobalt nitrate (Co(NO_3_)_2_·6H_2_O), 2-methylimidazole, anhydrous methanol (99.9%) (Sigma-Aldrich Company) were used to prepare ZIF-67 material. Furthermore, herbicide molinate (98% purity) (Kavosh Kimia Kerman Company) was used as an analyte.

### 2.2. Synthesis of MgAl_2_O_4_ nanoparticle

MgAl_2_O_4_ nanoparticles are prepared according to the reported method through an amended sol–gel route.^30^ In a typical preparation, (Al(NO_3_)_3_·9H_2_O) and (Mg(NO_3_)_2_·6H_2_O) with a molar proportion of 1 : 2 in addition to diethylene glycol and succinic acid with a molar proportion of 1 : 1 individually, were dissolved in distilled water. The solution was mixed and stirred for 1 h at 80 °C to form a sol. Then, in order to remove any excessive water, it was heated at 120 °C for 1 h. Afterward, the solution was heated for 1 h at 150 °C to form a dried gel. In the end, the resulting powders were calcined for 2 h at 800 °C.

### 2.3. Synthesis of ZIF-67@MgAl_2_O_4_ nanocomposite

0.21 g of MgAl_2_O_4_ and 0.87 g of Co(NO_3_)_2_·6H_2_O were dispersed in 60 mL of methanol solution under stirring for 1 h at room temperature. Simultaneously, a separate solution of 1.28 g of 2-methylimidazole in 60 mL of methanol was formed. Two solutions were slowly added to each other and stirred for 7 h at room temperature. Afterward, the gained solution washed two times with water and methanol, and it was dried under a vacuum for 10 h at 80 °C. The influence of weight of the different precursor materials on the reaction was studied as a variable parameter (Table 1). In this study, first, a higher weight percentage of ZIF-67 than MgAl_2_O_4_ spinel was used to make the composite (S_1_–S_3_ samples), then this percentage was equalized (S_4_ sample), and finally, the weight percentage of MgAl_2_O_4_ spinel to ZIF-67 was increased (S_5_ sample).

**Table 1:** The sample code table of as-prepared ZIF-67@MgAl_2_O_4_ nanocomposites at different weight ratios (S_1_–S_5_)

Sample code	MgAl_2_O_4_ (g)	(Co(NO_3_)_2_·6H_2_O) (g)	2-Methylimidazole (g)
S_1_	0.21	0.87	1.28
S_2_	0.51	0.82	1.23
S_3_	1.27	0.77	1.17
S_4_	1.44	0.52	0.92
S_5_	2.40	0.20	0.60

### 2.4. Characterization of samples

#### 2.4.1 Fourier transform infrared (FT-IR) spectroscopy

FT-IR spectra as a technique to recognize the chemical composition of ZIF-67@MgAl_2_O_4_ nanocomposites with a resolution of 4 cm^−1^ and the KBr pellet were registered in the 400–4000 cm^−1^ range (JASCO 680-PLUS spectrometer, Japan).

#### 2.4.2 X-ray diffraction (XRD)

Besides, the XRD patterns as a technique to characterization the phase structures with Cu Kα radiation (*λ* = 0.15406 nm) in the range of 2*θ* = 5–50° at a voltage of 40 kV and current of 30 mA were carried out by an X'Pert Pro-MPD equipment from Philips Company, USA.

#### 2.4.3 Field emission scanning electron microscope

The FESEM analysis as an identification technique of morphology and structure were detected *via* a Quanta FEG 450 device from FEI Company, USA.

#### 2.4.4 Brunauer–Emmett–Teller (BET)

The BET analysis (N_2_ adsorption–desorption at 273 K) as a technique to determination the textural properties was measured by a BELSORP-mini II equipment from MicrotracBEL Company, Japan.

#### 2.4.5 Thermogravimetric analysis (TGA)

Furthermore, the TGA analysis (under a nitrogen atmosphere, 15 degrees per minute) as a technique to investigation the thermal behavior was performed by a Pyris-1 equipment from PerkinElmer Company, USA.

#### 2.4.6 Inductively coupled plasma-optical emission spectrometry (ICP-OES)

In addition, the ICP-OES analysis as a technique to designation the composition of elements was used by a DV5311 model from PerkinElmer Company, USA.

#### 2.4.7 Corona discharge ionization-ion mobility spectrometry (CD-IMS)

Additionally, the CD-IMS as an detection technique was manufactured *via* Teif Azmon Espadana Co., (Isfahan, Iran) as beforehand explained in detail.^31–35^ The setup parameters of the CD-IMS apparatus were summarized in Table S1, in the ESI.†

### 2.5. Ion mobility spectrometry procedure

The principal part of the IMS, including IMS cell, pulse and two high voltage generators, an amplifier, a board control for converting the analog to digital signal and a computer. The IMS cell was manufactured from several conductive rings such as aluminum rings. Each ring was separated by isolate rings (*e.g.* PTFE). The conductive rings were linked by a resistor for creating the constant electric field. The IMS cell was formed from two regions, including reaction region (ionization source) and drift region. In this work, corona discharge (CD) was used for ionization of the sample molecules in the gaseous phase. Generally, corona discharge ionization is a sharp needle that is positioned about millimeters from a conductive plate with a high voltage of 1 to 4 kV between the needle and conductive electrode.^32^ For separating the reaction and drift regions, Bradbury–Nielsen grid is used. The ion gate was applied for pulse injecting the ionic samples to the drift region. In drift region, the ionic samples are moved to the detector (Faraday cup) based on the applied electric field (*E* = 200–500 V cm^−1^). The primary signal was formed by detector, then for improving the primary signal, an amplifier after detector was placed. Thereafter, the amplified signal was processed with analog to digital cards and shown by a computer. The signal for the analyte was plotted against the acquisition time, and the integration of this curve was considered as the peak area.

### 2.6. Evaluation of ZIF-67 performances as an adsorbent for the SPME method

#### 2.6.1 Solid-phase micro-extraction procedure

**Fig. 1:**
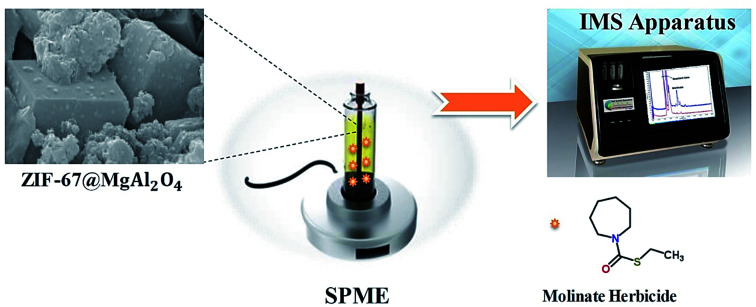
The schematic of the SPME procedure along with the analysis of molinate by CD-IMS device.

At first, for eliminating the Ni–Cr wire (as a SPME probe) pollution in the SPME method, the probe was placed in the methanol solvent. In addition, the solution of the silicone binder was prepared in the toluene solvent (purity of the prepared binder; 10% w/v) and the probe was placed in the binder solution. After 1 minute, the probe was exited from the binder solution and introduced to the ZIF-67@MgAl_2_O_4_ nanocomposite for 1 minute. Finally, in order to remove the toluene solvent and pollutants, the supported probe with ZIF-67@MgAl_2_O_4_ nanocomposite was placed at the temperature of 220 °C for 20 minutes. The extraction method along with the identification system was shown in the Fig. 1. The volume of the aqueous solution (10 mL) was transferred into the extraction cell (15 mL), afterward the extraction cell placed on a heater stirrer with the temperature of 5 °C. Thereupon, the ZIF-67@MgAl_2_O_4_ nanocomposite supported on the SPME wire was placed into the analyte aqueous solution (with pH = 2) to extract time of 30 min with stirrer rate of 600 rpm. After arriving at the equilibrium time between the adsorbent and aqueous solution, the composite wire was drawn back and rapidly introduced into the CD-IMS injection port (220 °C).

## 3. Results and discussion

### 3.1. Characteristics of ZIF-67@MgAl_2_O_4_ nanocomposite

**Fig. 2:**
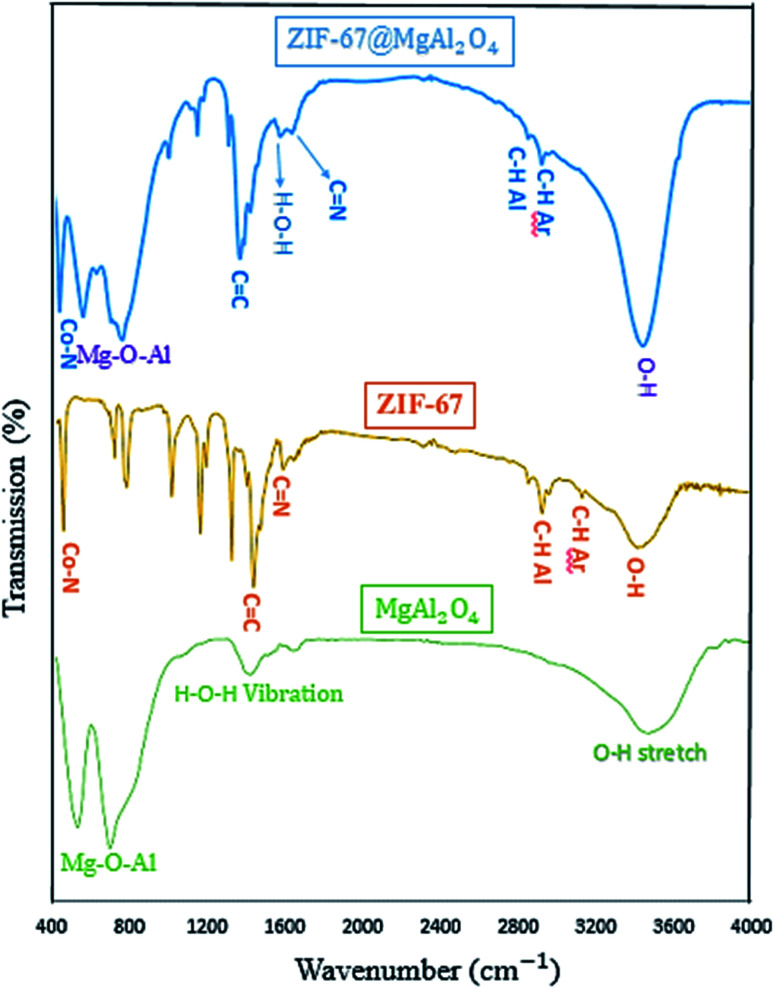
FT-IR spectra of MgAl_2_O_4_, ZIF-67 and ZIF-67@MgAl_2_O_4_.

The FT-IR spectra of MgAl_2_O_4_, ZIF-67, and ZIF-67@MgAl_2_O_4_ materials in the wavenumber range of 400–4000 cm^−1^ are presented in Fig. 2. The spectrum of MgAl_2_O_4_ indicate the presence of O–H stretching and H–O–H vibration. Furthermore, in the spectrum of the ZIF-67@MgAl_2_O_4_, the broad band at approximately 3460 cm^−1^ is assigned to the O–H stretching vibration.^33,34^ The bands at 3100 and 2900 cm^−1^ are ascribed to the aromatic and aliphatic C–H in the imidazole ring in case of ZIF-67.^18,21^ Besides, the band at 1619 cm^−1^ is related to the H–O–H vibration in case of MgAl_2_O_4_ and ZIF-67@MgAl_2_O_4_.^6,35^ The band at 1629 cm^−1^ is assigned to the C=N stretching vibration.^36,37^ Additionally, the band at 1420 cm^−1^ corresponds to the C=C bond in the imidazole ring in case of ZIF-67 and ZIF-67@MgAl_2_O_4_.^36–38^ The bands observed at about 540 and 700 cm^−1^ are associated with Mg–O–Al that show and confirm the presence of MgAl_2_O_4_.^11,34^ In addition, the band at 427 cm^−1^ is allocated to the Co–N band in case of ZIF-67 and ZIF-67@MgAl_2_O_4_.^37,39,40^

**Fig. 3:**
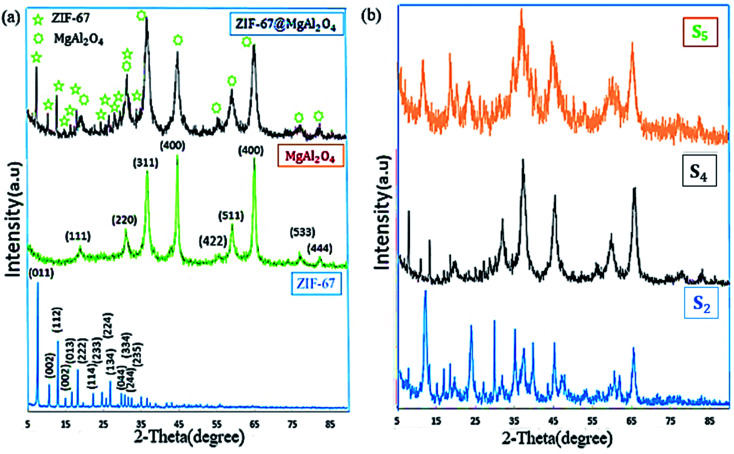
X-ray diffraction of (a) MgAl_2_O_4_, ZIF-67, ZIF-67@MgAl_2_O_4_ and (b) prepared composites in the different weight ratio were indicated as S_2_ (ZIF-67/MgAl_2_O_4_ = 4), S_4_ (ZIF-67/MgAl_2_O_4_ = 1) and S_5_ (ZIF-67/MgAl_2_O_4_ = 1/4) samples.

To characterize the phase structures of the as-synthesized ZIF-67@MgAl_2_O_4_ nanocomposite, XRD experiments were performed (Fig. 3). As displayed in Fig. 3a, the diffraction peaks of ZIF-67@MgAl_2_O_4_ nanocomposite at 2*θ* values of 19.1° (111), 31.3° (220), 36.8° (311), 45° (400), 55.7° (422), 59.2° (511), 65.4° (440), 77.4° (533) and 82.8° (444) consistent with those of the pure MgAl_2_O_4_ (JCPDS card no. 00-010-0062).^11,30^ X-ray diffraction patterns of ZIF-67@MgAl_2_O_4_ nanocomposite, showing that the combination of ZIF-67 has no remarkable influence on the crystal structure of MgAl_2_O_4_ nanoparticles. The peaks at 2*θ* values 7.31° (011), 10.36° (002), 12.72° (112), 14.40° (022), 16.45° (013), 18.04° (222), 22.15° (114), 24.53° (233), 25.62° (224), 26.70° (134), 29.67° (044), 30.62° (334), and 32.43° (235) match with the crystallographic structure of ZIF-67 reported formerly.^33,37^ Generally, two crystalline phases of ZIF-67 and MgAl_2_O_4_ are illustrated in the ZIF-67@MgAl_2_O_4_ nanocomposite. Additionally, the XRD pattern of the as-synthesized materials at different weight ratio were indicated in Fig. 3b. The patterns indicated that, with increasing the weight ratios of each of the precursors, the crystalline phase has been improved in the obtained ZIF-67@MgAl_2_O_4_ nanocomposite. The reason for the change in the XRD pattern could be that by increasing the weight ratio of ZIF-67 to MgAl_2_O_4_ spinel, the intensity of ZIF-67 peaks is much higher than MgAl_2_O_4_ spinel. As a result, some of the MgAl_2_O_4_ spinel peaks that are less intense are not visible. This also happens for the MgAl_2_O_4_ spinel, and as the weight ratio of spinel to ZIF-67 increases, a number of ZIF-67 peaks are not observed in the final pattern due to their lower intensity (although they are present). In the S_4_ sample, where the weight ratio of ZIF-67 and, MgAl_2_O_4_ spinel is the same, all peaks are seen in the XRD pattern of composite because the peaks are the same intensity.

**Fig. 4:**
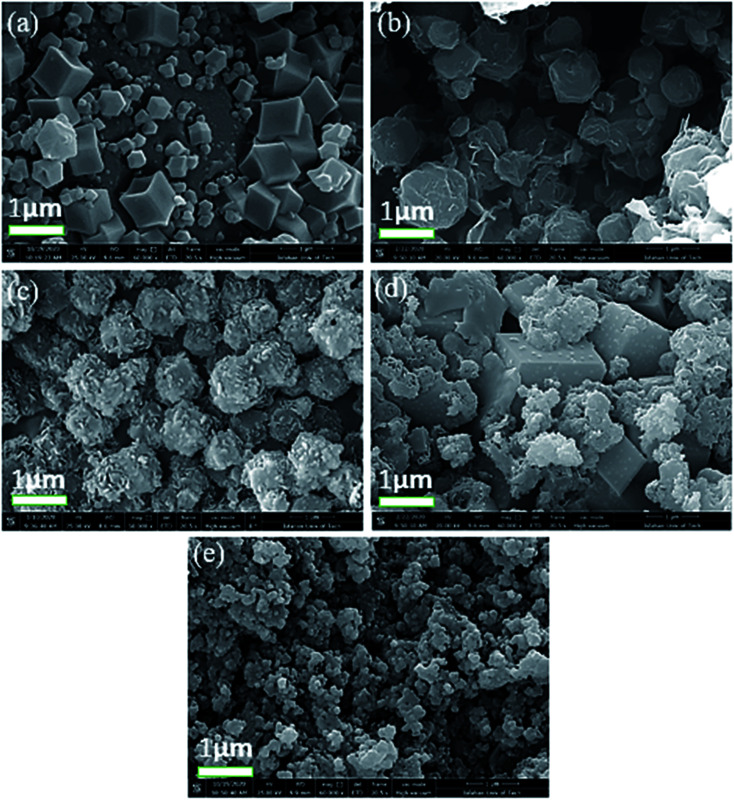
FESEM images of the synthesized composites with the magnification of 1 micrometer in the different weight ratio (a) S_1_ sample (ZIF-67/MgAl_2_O_4_ = 8), (b) S_2_ sample (ZIF-67/MgAl_2_O_4_ = 4), (c) S_3_ sample (ZIF-67/MgAl_2_O_4_ = 2), (d) S_4_ sample (ZIF-67/MgAl_2_O_4_ = 1) and (e) S_5_ sample (ZIF-67/MgAl_2_O_4_ = 1/4).

The morphologies of the prepared composite samples with the magnification of 1 micrometer at different weight ratios were studied by FE-SEM analysis (Fig. 4a–e). As displayed in Fig. 4a, rhombic dodecahedral morphology is observed for the S_1_ sample. The morphologic change is obtained by increasing the weight ratio of MgAl_2_O_4_ in the composite (indicate as S_2_, S_3_ and S_4_ sample) as shown in Fig. 4b–d. The average particle size of the as-synthesized MgAl_2_O_4_ nanoparticles are founded to be 35–50 nm. The leaf-like morphology with relatively uniform particles are obtained for these samples, and the unchanging constituent parts with quasi-spherical morphology are obtained for S_5_ samples (Fig. 4e). In addition, the particle size of S5 sample (ZIF-64/spinel = 1 : 4) was 80–90 nm. Generally, the cobalt ion first combines with the MgAl_2_O_4_ pre-preparing to form nucleation.^20^ Additionally, the images of the same samples but with different magnifications are founded in Fig. S1, ESI.†

**Fig. 5:**
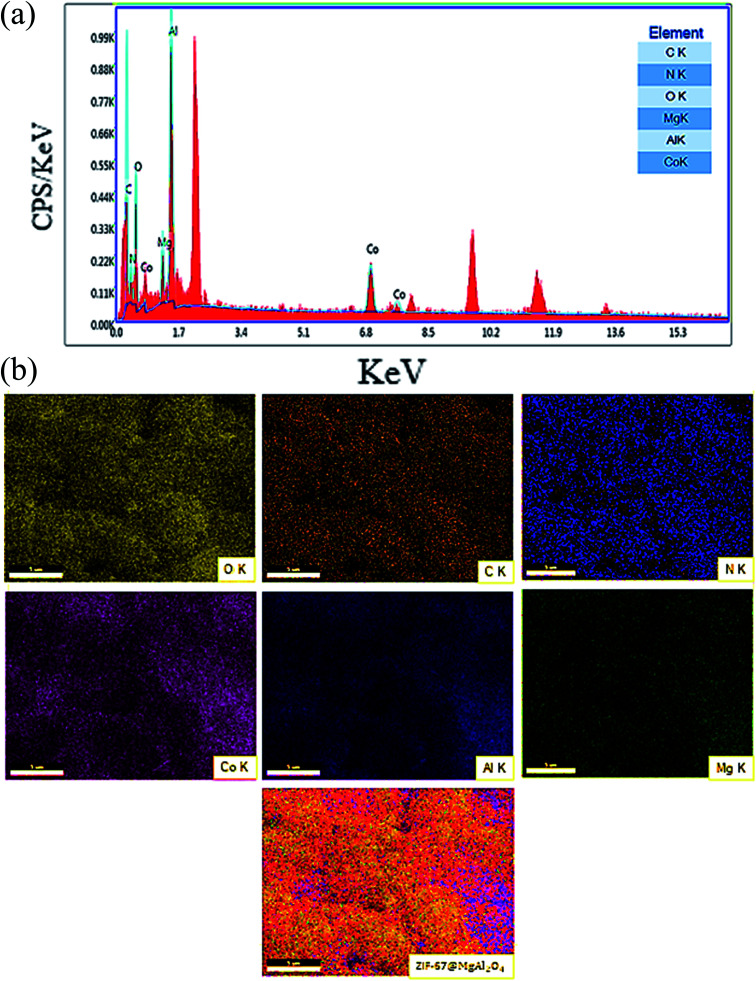
(a) EDS analysis and (b) X-ray mapping of ZIF-67@MgAl_2_O_4_ nanocomposite.

Consequently, the EDX analysis was checked to confirm the different elements in the composition of the ZIF-67@MgAl_2_O_4_ nanocomposite. The obtained result displayed the presence of C, N, O, Mg, Co and Al in the composite (Fig. 5a). As well, the existence of C, N, O, Mg, Co and Al elements in uniform distribution inside the ZIF-67@MgAl_2_O_4_ nanocomposite have been confirmed by X-ray mapping analysis (Fig. 5b).

ICP analysis was approved to detect the content of elements in the prepared composite.^41^ As displayed in Table 2, the content of Al^3+^, Mg^2+^ and Co^2+^ in the composite were 11.3 wt%, 4.55 wt% and 25.5 wt% respectively.

**Table 2:** ICP-OES analysis of ZIF-67@MgAl_2_O_4_ composite

Sample	Al^3+^ (wt%)	Mg^2+^ (wt%)	Co^2+^ (wt%)
ZIF-67@MgAl_2_O_4_	11.3	4.55	25.5

**Fig. 6:**
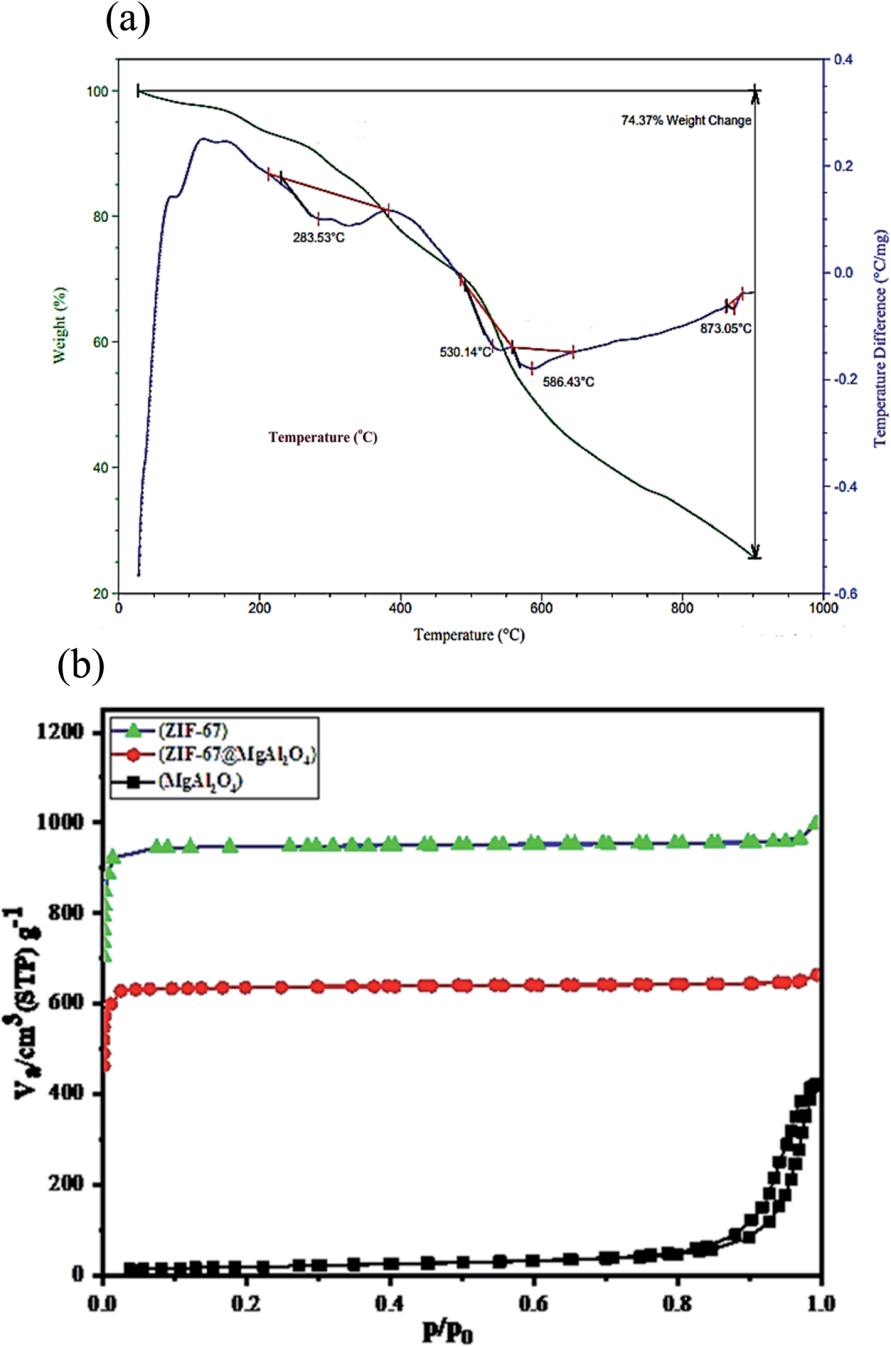
(a) TGA/DTA curves of ZIF-67@MgAl_2_O_4_ nanocomposite, (b) N_2_ adsorption/desorption isotherms of MgAl_2_O_4_, ZIF-67 and ZIF-67@MgAl_2_O_4_.

Additional description systems together with the TGA and DTA curves were tested for the prepared ZIF-67@MgAl_2_O_4_ nanocomposite materials (Fig. 6a). The total weight loss of the ZIF-67@MgAl_2_O_4_ composite is measured to be 74.4 wt%. Besides, the weight loss at 283 °C can be assigned to the escape of some species (*e.g.*, 2-methylimidazole) from the surfaces and guest molecules (*e.g.*, methanol) from the cavities of the composite. Furthermore, between 285 °C and 600 °C, ZIF-67@MgAl_2_O_4_ nanocomposite had a weight loss, which can be because of burning of the carbonaceous remnants of succinic acid and diethylene glycol, formation of γ-Al_2_O and thermal decomposition of the framework.^11,20,33,42^

The nitrogen adsorption–desorption analysis was checked to characterize the textural properties of the as-synthesized ZIF-67@MgAl_2_O_4_ nanocomposite. As indicated in Fig. 6b and Table 3, the specific surface area of ZIF-67@MgAl_2_O_4_ found to be 803.8 m^2^ g^−1^, while it was 64.8 m^2^ g^−1^ for MgAl_2_O_4_ material. The as-synthesized ZIF-67@MgAl_2_O_4_ displays a decreased specific surface area, compared with the ZIF-67 (1072.4 m^2^ g^−1^), which may originate from the heavier MgAl_2_O_4_ cores.^20,21,30^ According to the BET analysis, the pore size of ZIF-67@MgAl_2_O_4_ nanocomposite was founded to be 1.71 nm.

**Table 3:** Surface area of ZIF-67@MgAl_2_O_4_, ZIF-67 and MgAl_2_O_4_

Material	Specific surface area, m^2^ g^−1^
ZIF-67@MgAl_2_O_4_	803.8 ± 5^a^
ZIF-67	1072.4 ± 5
MgAl_2_O_4_	64.8 ± 5

a Standard deviation.

### 3.2. Performance of ZIF-67@MgAl_2_O_4_ nanocomposite

To evaluate the ability of ZIF-67@MgAl_2_O_4_ nanocomposite as an efficient adsorbent for solid phase microextraction method, three experiments were done when the MgAl_2_O_4_, ZIF-67, and ZIF-67@MgAl_2_O_4_ were used as the adsorbent for the SPME method. It is necessary to mention that three tests were at the same conditions (the aqueous solution volume; 10 mL, extraction temperature; 5 °C, sample pH; 2, extraction time; 30 min, and stirrer rate; 600 rpm). Firstly, 2 cm of Ni–Cr wire were selected and then MgAl_2_O_4_, ZIF-67, and ZIF-67@MgAl_2_O_4_ were separately coated on the Ni–Cr wire by silicone binder. Afterward, the adsorbents were used for extraction of molinate molecules (molinate concentration; 50 μg L^−1^). To detect the molinate molecule, the extracted molinate on the adsorbents were injected to the CD-IMS, respectively. The signals of IMS were plotted for comparing of three experiments. Fig. S2, ESI† depicts the CD-IMS spectra of molinate extracted of 50 μg L^−1^ by MgAl_2_O_4_, ZIF-67, and ZIF-67@MgAl_2_O_4_ in the SPME method. The peak areas of the extracted molinate were obtained 630, 1200, and 3500 for MgAl_2_O_4_, ZIF-67, and ZIF-67@MgAl_2_O_4_, respectively. Based on the calculated result, when the ZIF-67@MgAl_2_O_4_ was used as an adsorbent for SPME probe, the highest extraction efficiency was created. The reasons for the better adsorption of ZIF-67@MgAl_2_O_4_ nanocomposite than ZIF-67 and, MgAl_2_O_4_ spinel could be that each of the ZIF-67 and, MgAl_2_O_4_ spinel precursors alone perform specific interactions with the molinate molecule. But when they form a composite together, the existing interactions become more diverse and more numerous, thus increasing the adsorption property. According to the functional groups in the ZIF-67@MgAl_2_O_4_ surface and molinate structure, these interactions include hydrogen bonds, electrostatic interactions, and π interactions. While in MgAl_2_O_4_ and ZIF-67 alone the mentioned interactions are less and weaker. Also, in the SPME method, the insoluble properties and thermal stability of the prepared adsorbent are important. Therefore, in this research, spinel was used to improve the mentioned properties along with ZIF-67, which has a very good surface area.

### 3.3. Optimization of the parameters affected on the SPME method

#### 3.3.1 Extraction temperature and time

**Fig. 7:**
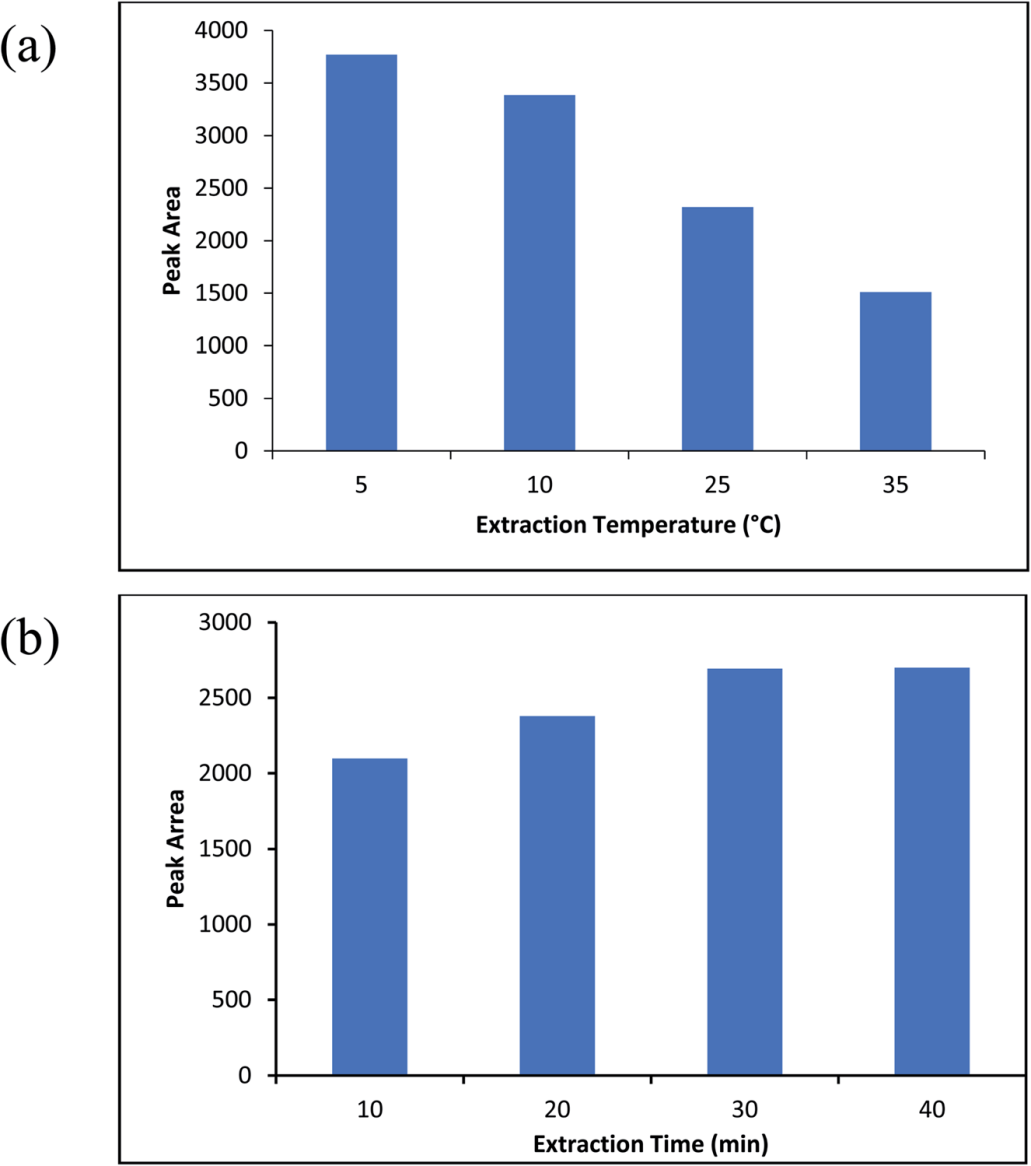
(a) The effect of extraction temperature, (b) the effect of extraction time on the extraction efficiency.

Temperature of the extraction solution is one of the effective parameters on the extraction efficiency. Based on the exothermic process for adsorption of the analyte molecule on the SPME wire, the increased temperature has an inverse effect on the extraction of the compound.^43^ To study the extraction temperature, some experiments (sample volume; 10 mL, the molinate concentration; 50 μg L^−1^) were carried out at the temperatures of 5–45 °C. Based on the obtained result that indicated in Fig. 7a, 5 °C was designated as the optimized point for the next experiments. Further, to arrive of the equilibrium time between aqueous samples and ZIF-67@MgAl_2_O_4_ in the SPME technique,^44^ the extraction time between 10–40 min was examined (sample volume; 10 mL, the molinate concentration; 50 μg L^−1^). Fig. 7b indicates the best extraction efficiency for analysing of the molinate at the time of 30 min.

#### 3.3.2 Stirring rate and sample pH

**Fig. 8:**
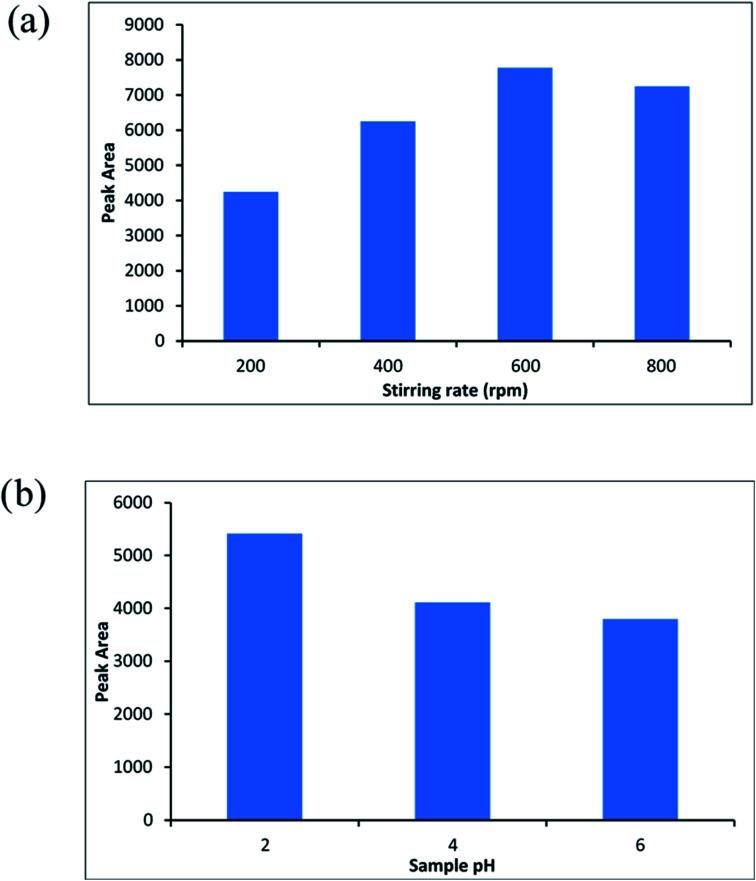
(a) The effect of stirring rate, (b) the effect of sample pH on the extraction efficiency.

The mass transfer process of the molinate from the sample solution to the adsorbent is related to the sample agitation. In this regard, the solution of analyte (sample volume; 10 mL, the molinate concentration; 50 μg L^−1^) was stirred at 200–800 rpm. Fig. 8a shows the effect of stirring rate on the extraction efficiency as the highest efficiency of the method was related to 600 rpm.

One of the important conditions for extracting of the analyte from aqueous solution is the molinate that should be in the neutral shape. Therefore, the pH of the sample is one of the most important parameters that affect the extraction efficiency. Accordingly, sample pH of 2 until 6 were investigated and optimized (sample volume; 10 mL, the molinate concentration was about 50 μg L^−1^). Fig. 8b indicates the effect of sample pH on the efficiency of the method and pH = 2 was chosen as an optimized point.

### 3.4. Analytical parameters

For checking the ability of the proposed method, some analytical parameters, such as the limit of quantification (LOQ), method detection limit (MDL), linear dynamic range (LDR) with correlation coefficient (*R*^2^) were appraised with the SPME method for extracting of molinate molecules and then identification by CD-IMS apparatus. In this regard, different molinate concentrations (10 to 150 μg L^−1^) were prepared in the water solvent. The molinate molecules were extracted by SPME method and injected to the CD-IMS. By the least square method, the calibration curve was plotted and according to the S/N = 3 and S/N = 10, the MDL and LOQ were acquired 3 and 10 μg L^−1^, respectively. The molinate concentration range of 10 to 100 μg L^−1^ was obtained as the LDR of the method and the *R*^2^ value was calculated 0.9961. Generally, in the IMS technique, the sensitivity and selectivity are introduced to signal to noise ratio (S/N) and the drift time, respectively. The relative standard deviation (RSD%) of the ZIF-67@MgAl_2_O_4_ as an adsorbent for extracting the molinate at the concentration of 50 μg L^−1^ was calculated 4%. The enrichment factor (EF) was calculated by dividing the final concentration of the molinate (the extracted molinate concentration from a spiked sample) to the primary molinate concentration. In this method the EF was obtained 5.

### 3.5. Method comparison

Table 4 displays a comparison of some analytical parameters reached in the study with those reported by other studies for the determination of herbicide molinate. The method presented in the present study to measure herbicide molinate is a simple technique with a suitable detection limit. Despite the availability of the robust separation systems, including high-performance liquid chromatography (HPLC) or gas chromatography (GC) it is almost known as an expensive technique with high run time. On the other hand, the detectors of the HPLC or GC have been used for special compounds not all compounds and accordingly some compounds should be derivatized and so the time and cost are increased severely. The IMS is used for analysing of the various compounds without limitations such as derivatization or high run time. However, in the complex matrices, a stand-alone IMS is not known as a powerful identification system and need to combine with the separation system (HPLC or GC) or sample preparation methods. In the HMDE-based method, mercury is poisonous and it should be definitely shunned for analytical goals. According to the functional groups in the ZIF-67@MgAl_2_O_4_ surface and molinate structure, the salt bridge interaction is performed for adsorbing of the molinate. In addition, it has contained electrostatic interaction and hydrogen bonds between adsorbent and analyte.

**Table 4:** Comparison of MLD and LDR reached *via* the technic with those reported by other

Method	Sample type	MDL^a^ (mg L^−1^)	LDR^b^ (mg L^−1^)	Reference
GC-MS-MS	Water	4 (μg L^−1^)	—	45
Voltammetric-HMDE^d^	Water	6.6 (μg L^−1^)	0.94–1.69	46
HPLC	Water	0.9	0.9–37.5	47
GC-FPD	Water	2.83 (μg L^−1^)	—	48
GST-based biosensor	Water	0.064	19–7.9	49
SPME-CD-IMS^c^	Water	3 (μg L^−1^)	10–100 (μg L^−1^)	This work

a Method detection limit.

b Linear dynamic range.

c Solid phase microextraction-corona discharge-ion mobility spectrometer.

d HMDE—hanging mercury drop electrode.

## 4. Conclusions

In summary, ZIF-67@MgAl_2_O_4_ nanocomposite was successfully prepared for the first time at room temperature *via* a novel technique with diverse morphologies. To approve the structural characterization of ZIF-67@MgAl_2_O_4_ nanocomposite different techniques like TGA, XRD, BET and FE-SEM were applied. Furthermore, to investigate the capability of the ZIF-67@MgAl_2_O_4_ as an adsorbent, solid phase microextraction method was applied to extract the herbicide molinate (as a test compound) in the aqueous samples. Extraction time and temperature were optimized 30 min and 5 °C respectively. The dynamic range (LDR) of the altered concentrations of the molinate and correlation coefficient were 10.0–100.0 μg L^−1^ and 0.9961, respectively. In addition, limit of quantification (LOQ) and method detection limit (MDL) were obtained 10.0 μg L^−1^ and 3.0 μg L^−1^, respectively. In this research, the adsorption behaviour of ZIF-67@MgAl_2_O_4_ nanocomposite was investigated and this composite has different applications due to its different properties, which can be studied in future studies.

## Author contributions

M. D. helped in preparing the material, characterization, and writing the manuscript. M. R. R. and M. T. J. helped in characterization of the obtained materials as well as the discussion of the obtained results. Furthermore, F. D., M. B. and A. E. S. designed the research, contributed to supervising the work, discussed the results, and wrote the manuscript. All the authors participated in writing, editing, and revising the manuscript.

## Conflicts of interest

The authors declare no conflict of interest.

## Supplementary Material

RA-011-D1RA01056E-s001

## References

[cit1] Sancho E., Cerón J. J., Ferrando M. D. (2000). Ecotoxicol. Environ. Saf..

[cit2] Nunes O. C., Lopes A. R., Manaia C. M. (2013). Appl. Microbiol. Biotechnol..

[cit3] Cohen M. J., Karasek F. W. (1970). J. Chromatogr. Sci..

[cit4] Borsdorf H., Eiceman G. A. (2006). Appl. Spectrosc. Rev..

[cit5] Ganesh I. (2013). Int. Mater. Rev..

[cit6] Habibi N., Wang Y., Arandiyan H., Rezaei M. (2017). Adv. Powder Technol..

[cit7] Baudín C., Martínez R., Pena P. (1995). J. Am. Ceram. Soc..

[cit8] Sindel M., Travitzky N. A., Claussen N. (1990). J. Am. Ceram. Soc..

[cit9] Ganesh I. (2002). et al.. Ceram. Int..

[cit10] Saberi A., Golestani-Fard F., Willert-Porada M., Simon R., Gerdes T., Sarpoolaky H. (2008). J. Eur. Ceram. Soc..

[cit11] Sanjabi S., Obeydavi A. (2015). J. Alloys Compd..

[cit12] Schmidt-Verma A. K. (2020). et al.. Adv. Eng. Mater..

[cit13] Jian M., Liu B., Liu R., Qu J., Wang H., Zhang X. (2015). RSC Adv..

[cit14] Taheri-Ledari R., Mirmohammadi S. S., Valadi K., Maleki A., Shalan A. E. (2020). RSC Adv..

[cit15] Wang B., Côté A. P., Furukawa H., O'Keeffe M., Yaghi O. M. (2008). Nature.

[cit16] Achmann S., Hagen G., Kita J., Malkowsky I. M., Kiener C., Moos R. (2009). Sensors.

[cit17] Kim J., Kim S.-N., Jang H.-G., Seo G., Ahn W.-S. (2013). Appl. Catal., A.

[cit18] Mostafazadeh N., Ghoreyshi A. A., Pirzadeh K. (2018). Iran. J. Chem. Chem. Eng..

[cit19] Xu G., Yamada T., Otsubo K., Sakaida S., Kitagawa H. (2012). J. Am. Chem. Soc..

[cit20] Yang Q. (2018). et al.. Chem. Eng. J..

[cit21] Barjola A., Escorihuela J., Andrio A., Giménez E., Compañ V. (2018). Nanomaterials.

[cit22] Jiang Z., Li Z., Qin Z., Sun H., Jiao X., Chen D. (2013). Nanoscale.

[cit23] Kasik A., Dong X., Lin Y. S. (2015). Microporous Mesoporous Mater..

[cit24] Drobek M. (2015). et al.. J. Membr. Sci..

[cit25] Guo X., Xing T., Lou Y., Chen J. (2016). J. Solid State Chem..

[cit26] Koh K., Wong-Foy A. G., Matzger A. J. (2009). Chem. Commun..

[cit27] Ke F., Qiu L. G., Yuan Y. P., Jiang X., Zhu J. F. (2012). J. Mater. Chem..

[cit28] Yang J. (2015). et al.. Angew. Chem..

[cit29] Della Rocca J., Liu D., Lin W. (2011). Acc. Chem. Res..

[cit30] Karami N., Davar F., Hassani S. (2019). Mater. Res. Express.

[cit31] Rezayat M. R., Jafari M. T. (2020). Microchem. J..

[cit32] Eiceman G. A. and Karpas Z., Ion Mobility Spectrometry, CRC Press, Boca Raton, FL, 2nd edn, 2005

[cit33] Zhang Z., Zhang J., Liu J., Xiong Z., Chen X. (2016). Water, Air, Soil Pollut..

[cit34] Singh V., Chakradhar R. P. S., Rao J. L., Kim D. K. (2007). J. Solid State Chem..

[cit35] Guo J., Lou H., Zhao H., Wang X., Zheng X. (2004). Mater. Lett..

[cit36] Cravillon J., Nayuk R., Springer S., Feldhoff A., Huber K., Wiebcke M. (2011). Chem. Mater..

[cit37] Davoodi M., Davar F., Rezayat M. R., Jafari M. T., Shalan A. E. (2021). RSC Adv..

[cit38] Ulu A. (2020). J. Mater. Sci..

[cit39] Li X., Gao X., Ai L., Jiang J. (2015). Chem. Eng. J..

[cit40] Liu H. (2019). et al.. J. Mater. Res. Technol..

[cit41] Anna T., Sandri M., Landi E., Pressato D., Francioli S., Quarto R., Martin I. (2008). Biomaterials.

[cit42] Janosch C., Münzer S., Lohmeier S., Feldhoff A., Huber K., Wiebcke M. (2009). Chem. Mater..

[cit43] Wan Ibrahim W. A., Farhani H., Sanagi M. M., Aboul-Enein H. Y. (2010). J. Chromatogr. A.

[cit44] Jafari M. T., Rezayat M. R., Mossaddegh M. (2018). Talanta.

[cit45] Penetra A., Vale Cardoso V., Ferreira E., Benoliel M. J. (2010). Water Sci. Technol..

[cit46] Barroso M. F., Nunes O. C., Vaz M. C., Delerue-Matos C. (2005). Anal. Bioanal. Chem..

[cit47] Barreiros L., Manaia C. M., Nunes O. C. (2011). Biodegradation.

[cit48] Castro M., Silva-Ferreira A. C., Manaia C. M., Nunes O. C. (2005). Chemosphere.

[cit49] Oliveira T. I. S. (2013). et al.. Talanta.

