# Correction: Synthesis and characterization of a new ZIF-67@MgAl_2_O_4_ nanocomposite and its adsorption behaviour

**DOI:** 10.1039/d1ra90165f

**Published:** 2021-10-28

**Authors:** Mehdi Davoodi, Fatemeh Davar, Mohammad R. Rezayat, Mohammad T. Jafari, Mehdi Bazarganipour, Ahmed Esmail Shalan

**Affiliations:** Department of Chemistry, Isfahan University of Technology Isfahan 84156-83111 Iran davar@cc.iut.ac.ir; BCMaterials, Basque Center for Materials, Applications and Nanostructures, Martina Casiano, UPV/EHU Science Park Barrio Sarriena s/n Leioa 48940 Spain a.shalan133@gmail.com ahmed.shalan@bcmaterials.net; Central Metallurgical Research and Development Institute (CMRDI) P.O. Box 87, Helwan Cairo 11421 Egypt; Research Institute for Nanotechnology and Advanced Materials, Isfahan University of Technology Isfahan 84156-83111 Iran

## Abstract

Correction for ‘Synthesis and characterization of a new ZIF-67@MgAl_2_O_4_ nanocomposite and its adsorption behaviour’ by Mehdi Davoodi *et al.*, *RSC Adv.*, 2021, **11**, 13245–13255, DOI: 10.1039/D1RA01056E.

The authors regret that an incorrect affiliation was given for co-author Mehdi Bazarganipour in the original article. The correct affiliations are as shown here.

The Royal Society of Chemistry apologises for these errors and any consequent inconvenience to authors and readers.

## Supplementary Material

