# Expression of concern: Synthesis and characterization of a new ZIF-67@MgAl_2_O_4_ nanocomposite and its adsorption behaviour

**DOI:** 10.1039/d3ra90113k

**Published:** 2023-11-08

**Authors:** Mehdi Davoodi, Fatemeh Davar, Mohammad R. Rezayat, Mohammad T. Jafari, Mehdi Bazarganipour, Ahmed Esmail Shalan

**Affiliations:** a Department of Chemistry, Isfahan University of Technology Isfahan 84156-83111 Iran davar@cc.iut.ac.ir; b Nanotechnology and Advanced Material Institute, Isfahan University of Technology Isfahan 84156-83111 Iran; c BCMaterials, Basque Center for Materials, Applications and Nanostructures, Martina Casiano, UPV/EHU Science Park Barrio Sarriena s/n Leioa 48940 Spain a.shalan133@gmail.com ahmed.shalan@bcmaterials.net; d Central Metallurgical Research and Development Institute (CMRDI) P. O. Box 87, Helwan Cairo 11421 Egypt

## Abstract

Expression of concern for ‘Synthesis and characterization of a new ZIF-67@MgAl_2_O_4_ nanocomposite and its adsorption behaviour’ by Mehdi Davoodi *et al.*, *RSC Adv.*, 2021, **11**, 13245–13255, https://doi.org/10.1039/D1RA01056E

The Royal Society of Chemistry is publishing this Expression of concern in order to alert readers that concerns have been raised regarding the reliability of the SEM data in Fig. S1b and d. An investigation is underway, and an Expression of concern will continue to be associated with the article until a final outcome is reached.

Laura Fisher

2nd November 2023

Executive Editor, *RSC Advances*

## Supplementary Material

