# Supplementary material for: Synthesis and characterization of a new ZIF-67@MgAl2O4 nanocomposite and its adsorption behaviour
Source: RSC Adv. 2021 Apr 8;11(22):13245–55. doi: 10.1039/d1ra01056e (PMC8697589; doi:10.1039/d1ra01056e)
Supplement: RA-011-D1RA01056E-s001 [file RA-011-D1RA01056E-s001.pdf]

Electronic Supplementary Material (ESI) for RSC Advances.
This journal is © The Royal Society of Chemistry 2021

Supporting information

Synthesis and characterization of a new ZIF-67@$MgAl_2O_4$ composite and its adsorption behaviour

Mehdi Davoodi[a], Fatemeh Davar[a,], Mohammad R. Rezayat[a], Mohammad T. Jafari[a] ,*

Mehdi Bazarganipour, and Ahmed Esmail Shalan[b, c,,+]*

[a] Department of Chemistry, Isfahan University of Technology, Isfahan, 84156-83111, Iran

[b] BCMaterials, Basque Center for Materials, Applications and Nanostructures, Martina Casiano, UPV/EHU Science Park, Barrio Sarriena s/n, Leioa 48940, Spain.

[c] Central Metallurgical Research and Development Institute (CMRDI), P.O. Box 87, Helwan, Cairo 11421, Egypt.

**Corresponding authors. E-mail addresses: davar@cc.iut.ac.ir (F. Davar), a.shalan133@gmail.com (A. E. Shalan).*

[+] Currently on leave from CMRDI

Table S1: The operation conditions of the CD-IMS apparatus.

Parameter	Setting
IMS mode	Positive
IMS type	Drift tube
Ionization source	Corona discharge
IMS detector	Faraday cup
Needle voltage	2.0 kV
Target electrode voltage	7.0 kV
Drift electric field	400 V cm^{-1}
Drift gas flow (N_2, 99.999%)	1000 mL min^{-1}
Carrier gas flow (N_2, 99.999%)	800 mL min^{-1}
IMS cell temperature	150 ºC
Injection port temperature	220 ºC
Drift tube length	11 cm
Shutter grid pulse	180 µs
Shutter grid voltage	200 V
Shutter grid frequency	25 Hz
Number of IMS averages	25
Number of points per ion mobility spectrum	500

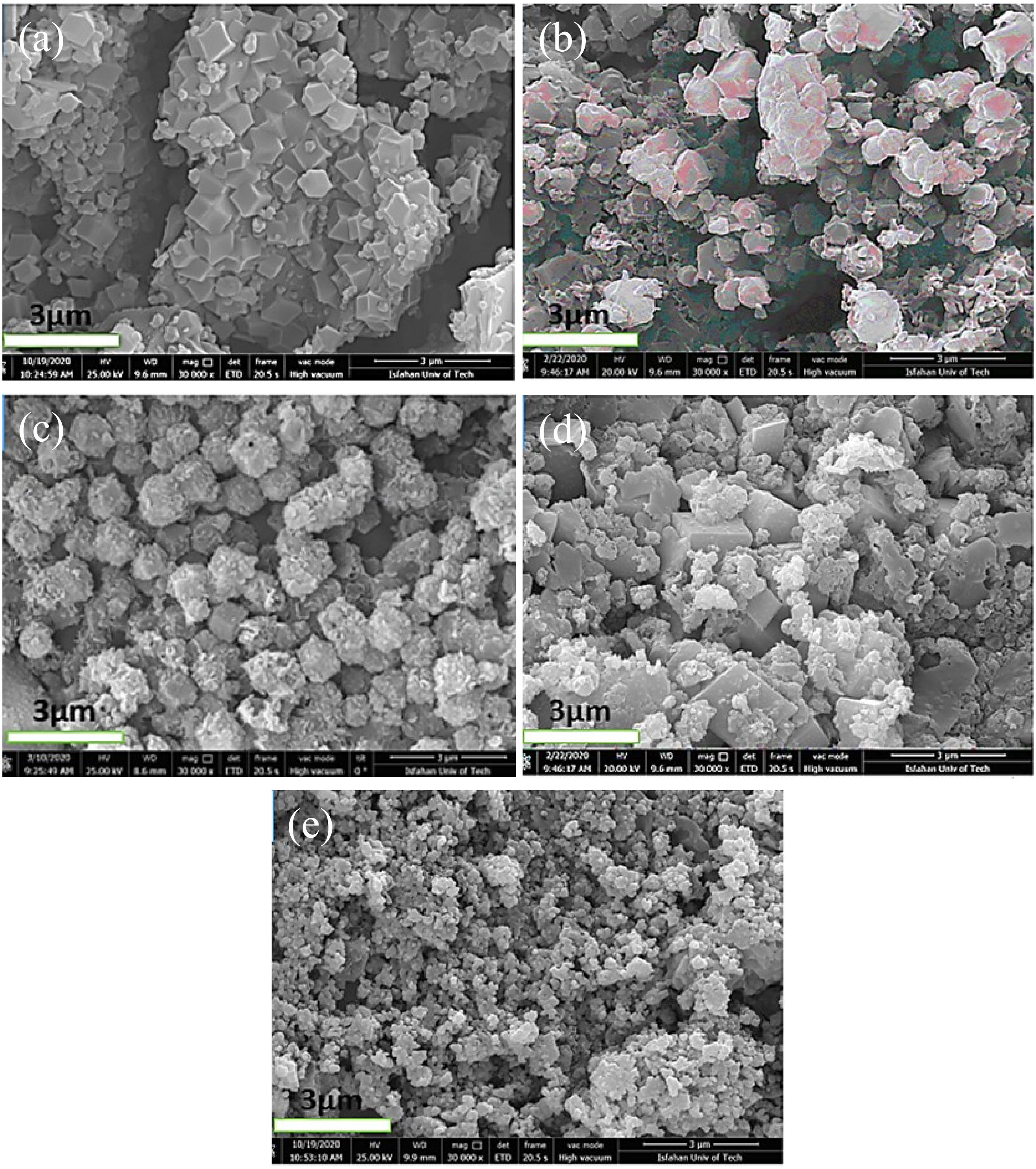

Figure S1: FE-SEM images of the synthesized composites with the magnification of 3 micrometre in the different weight ratio a) S_1 sample (*ZIF-67/MgAl$_2$O$_4$ = 8*), b) S_2 sample (*ZIF-67/MgAl$_2$O$_4$ = 4*), c) S_3 sample (*ZIF-67/MgAl$_2$O$_4$ = 8*), d) S_4 sample (*ZIF-67/MgAl$_2$O$_4$ = 8*), and d) S_5 sample (*ZIF-67/MgAl$_2$O$_4$ = 8*).

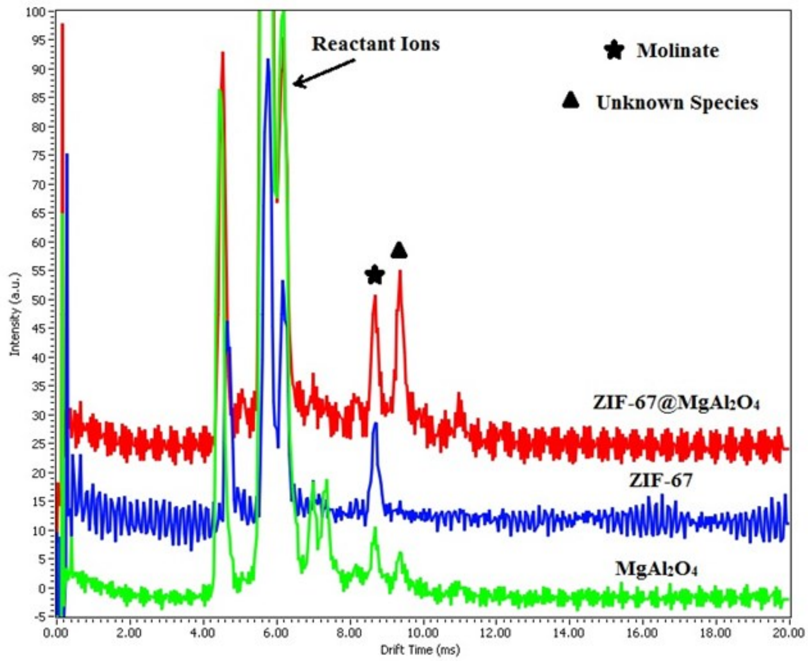

Figure S2: Ion mobility spectra of the molinate extracted by $MgAl_2O_4$, ZIF-67, and ZIF-67@$MgAl_2O_4$, (sample volume, 10 mL; molinate concentration; 50 µg L^{-1}).
